# CSI Pollen: Diversity of Honey Bee Collected Pollen Studied by Citizen Scientists

**DOI:** 10.3390/insects12110987

**Published:** 2021-11-02

**Authors:** Robert Brodschneider, Elfriede Kalcher-Sommersguter, Sabrina Kuchling, Vincent Dietemann, Alison Gray, Janko Božič, Andrejs Briedis, Norman L. Carreck, Robert Chlebo, Karl Crailsheim, Mary Frances Coffey, Bjørn Dahle, Amelia Virginia González-Porto, Janja Filipi, Dirk C. de Graaf, Fani Hatjina, Pavlos Ioannidis, Nicoleta Ion, Asger Søgaard Jørgensen, Preben Kristiansen, Antoine Lecocq, Jean-François Odoux, Asli Özkirim, Magnus Peterson, Blaž Podrižnik, Slađan Rašić, Gina Retschnig, Aygün Schiesser, Simone Tosi, Flemming Vejsnæs, Geoffrey Williams, Jozef J.M. van der Steen

**Affiliations:** 1 Institute of Biology, University of Graz, Universitätsplatz 2, 8010 Graz, Austria; elfriede.kalcher@uni-graz.at (E.K.-S.); karl.crailsheim@uni-graz.at (K.C.); 2 Austrian Agency for Health and Food Safety (AGES) GmbH, Data, Statistics and Integrative Risk Assessment, 8010 Graz, Austria; sabrina.kuchling@ages.at; 3 Swiss Bee Research Center, Agroscope, Schwarzenburgstrasse 161, 3003 Bern, Switzerland; vincent.dietemann@agroscope.admin.ch (V.D.); grw0010@auburn.edu (G.W.); 4 Department of Ecology and Evolution, Biophore, UNIL-Sorge, University of Lausanne, 1015 Lausanne, Switzerland; 5 Department of Mathematics and Statistics, University of Strathclyde, Livingstone Tower, 26 Richmond Street, Glasgow G1 1XH, UK; a.j.gray@strath.ac.uk (A.G.); magnuspeterson77@gmail.com (M.P.); 6 Department of Biology, Biotechnical Faculty, University of Ljubljana, Večna pot 111, 1000 Ljubljana, Slovenia; janko.bozic@bf.uni-lj.si (J.B.); blaz.podriznik@gmail.com (B.P.); 7 Latvian Beekeepers Association, Rīgas iela 22, LV-3004 Jelgava, Latvia; andrejs.briedis@inbox.lv; 8 Carreck Consultancy Ltd., Woodside Cottage, Dragons Lane, Shipley RH13 8GD, UK; norman.carreck@btinternet.com; 9 Laboratory of Apiculture and Social Insects, School of Life Sciences, University of Sussex, Brighton BN1 9QG, UK; 10 Institute of Animal Husbandry, Slovak University of Agriculture, Tr. A. Hlinku 2, 949 01 Nitra, Slovakia; robert.chlebo@uniag.sk; 11 Department of Agriculture Food & Marine, Backweston Laboratory Campus, Celbridge, W23 X3PH Co. Kildare, Ireland; maryf.coffey@agriculture.gov.ie; 12 Norwegian Beekeepers Association, Dyrskuev 20, 2040 Kløfta, Norway; bjorn.dahle@norbi.no; 13 Centro Agrario de Marchamalo-IRIAF, Camino San Martin s/n, 19180 Guadalajara, Spain; avgp2006@hotmail.com; 14 Department of Ecology, Agronomy and Aquaculture, University of Zadar, 23000 Zadar, Croatia; jfilipi@unizd.hr; 15 Honeybee Valley, Ghent University, Krijgslaan 281 S2, B-9000 Ghent, Belgium; Dirk.deGraaf@UGent.be; 16 Department of Apiculture, Institute of Animal Science, Hellenic Agricultural Organization ‘DEMETER’, 63200 Nea Moudania, Greece; fhatjina@gmail.com (F.H.); pionhelvet@windowslive.com (P.I.); 17 Beekeeping Research and Development Institute, 011464 Bucharest, Romania; ionnicoleta2006@yahoo.com; 18 Danish Beekeepers Association, 4180 Sorø, Denmark; asj@biavl.dk (A.S.J.); fv@biavl.dk (F.V.); 19 Apinordica AB, 59190 Tjaellmo, Sweden; preben.kristiansen@apinordica.se; 20 Department of Plants and Environmental Sciences—PLEN, University of Copenhagen, Thorvaldsensvej 40, 1871 Frederiksberg C, Denmark; antoine@plen.ku.dk; 21 Équipe “Écologie des Prairies”, UMR INRAE-UNICAEN 950 EVA, Université de Caen, Esplanade de la Paix, CEDEX 42, 14032 Caen, France; jean-francois.odoux@inrae.fr; 22 INRAE-APIS UE1255, Le Magneraud, 17700 Surgères, France; 23 Bee Health Laboratory, Department of Biology, The Beytepe Campus, Hacettepe University, Ankara 06800, Turkey; asli.ozkirim@gmail.com (A.Ö.); aygun@hacettepe.edu.tr (A.S.); 24 Faculty of Ecological Agriculture, EDUCONS University, 21208 Sremska Kamenica, Serbia; rasic.sladjan@gmail.com; 25 Institute of Bee Health, Vetsuisse Faculty, University of Bern, Schwarzenburgstrasse 161, 3003 Bern, Switzerland; gina.retschnig@vetsuisse.unibe.ch; 26 Department of Agricultural, Forest and Food Sciences, University of Turin, 10095 Turin, Italy; simone.tosi@unito.it; 27 Entomology & Plant Pathology, Auburn University, Auburn, AL 36849, USA; 28 Plant Research International, Wageningen UR, 6708 PB Wageningen, The Netherlands; alveusab@outlook.com; 29 Alveus AB Consultancy, 5061 EL Oisterwijk, The Netherlands

**Keywords:** *Apis mellifera*, citizen science, foraging ecology, nutrition, diversity, landscape, season, COLOSS

## Abstract

**Simple Summary:**

Honey bee colonies collect pollen from plants as a source of nutrients. Diverse diets comprising pollen from many different plant species are beneficial for honey bee colony health, because they contain a greater diversity of nutrients than monofloral diets of one plant species only. Here, we present the results of the COLOSS “CSI Pollen” study on the diversity of pollen collected by honey bee colonies. In this study, beekeepers acted as citizen scientists sampling and analyzing pollen collected by their own colonies. As a simple measure of diversity, beekeepers determined the number of different colors found in pollen samples that were collected in a coordinated and standardized way. The support of 750 beekeepers allowed the collection of information about almost 18,000 pollen samples from many European countries. We found that the pollen samples consistently comprised approximately six different colors in total, of which four colors were abundant. ‘Urban’ habitats or ‘artificial surfaces’ were associated with higher pollen color diversity. This investigation highlights seasonal- and land-use-related differences in the pollen supply for honey bees, which affects beekeeping and pollinator health. Determining pollen colors is a simple, useful technique for beekeepers to estimate pollen diversity.

**Abstract:**

A diverse supply of pollen is an important factor for honey bee health, but information about the pollen diversity available to colonies at the landscape scale is largely missing. In this COLOSS study, beekeeper citizen scientists sampled and analyzed the diversity of pollen collected by honey bee colonies. As a simple measure of diversity, beekeepers determined the number of colors found in pollen samples that were collected in a coordinated and standardized way. Altogether, 750 beekeepers from 28 different regions from 24 countries participated in the two-year study and collected and analyzed almost 18,000 pollen samples. Pollen samples contained approximately six different colors in total throughout the sampling period, of which four colors were abundant. We ran generalized linear mixed models to test for possible effects of diverse factors such as collection, i.e., whether a minimum amount of pollen was collected or not, and habitat type on the number of colors found in pollen samples. To identify habitat effects on pollen diversity, beekeepers’ descriptions of the surrounding landscape and CORINE land cover classes were investigated in two different models, which both showed that both the total number and the rare number of colors in pollen samples were positively affected by ‘urban’ habitats or ‘artificial surfaces’, respectively. This citizen science study underlines the importance of the habitat for pollen diversity for bees and suggests higher diversity in urban areas.

## 1. Introduction

A honey bee colony is a long-lived superorganism whose survival depends on pollen and nectar provided by flowers. In principle, a wide range of plant species are available to forage on, closely connected with season and landscape composition [1,2,3,4]. Despite being a polylectic organism, the honey bee colony does not collect pollen from all flowers available but instead concentrates on a selection of plant species [1,5]. Foragers do not seem to be able to evaluate the nutritional value of pollen during collection; they simply collect and transport. The nutritional value of pollen shows afterwards in the colony [6,7].

The chemical composition of bee-collected pollen loads has been well investigated to understand its importance for honey bee nutrition, but also for commercial purposes [8,9,10,11]. The biochemical composition of pollen from different plant species, and hence its nutritional value is dependent on landscape factors such as soil type and climate and varies throughout a season [12]. Polylectic pollen collection and hence consumption of a highly diverse diet is regarded as more beneficial for honey bees than monofloral pollen diets [13,14,15,16,17,18]. Pollen is diverse in its proportion of nutrients for honey bees, so a generalist collection strategy ensures the bees consume a diverse range of nutrients [13,19,20,21,22]. There is thus a spatial and temporal aspect of pollen diversity for the honey bee: spatial due to the pollen available in the foraging area and temporal due to the change of flowering patterns in the various landscapes over the season.

Landscape composition and habitat result in environments with greatly varying plant biodiversity and hence value to honey bees [12,23]. During the last century, there have been extensive changes in land use, which has affected bees [24,25,26,27,28,29]. Loss of semi-natural vegetation and increase in surface area of crop monocultures affect the quality and availability of food sources for honey bee colonies due to a reduction in plant diversity [30,31]. Goulson et al. [32] and many other researchers have blamed this reduction, among other factors such as pesticides and parasites, to be crucial for bee health. Reconstruction of native habitat and a more continuous supply of floral resources in large agricultural landscapes could partially mitigate this [29,33,34,35].

Information on plant diversity available to the honey bee can be obtained indirectly from pollen in honey (melissopalynology), directly from the pollen grains found on the bee’s hairs, from beebread (pollen stored in the comb), or from bee-collected pollen loads sampled using pollen traps placed at the entrance of the hive. Honey is the result of mixtures of nectars collected and mixed over a long period. Pollen found in honey thus summarizes a whole foraging season and may include pollen that has not been collected by foragers in their corbiculae (pollen baskets), but that was incidentally deposited on the bees’ surface during nectar collection, or pollen from wind pollinated plants that may land on flowers [36,37,38,39]. On the other hand, the pollen stripped from a bee using a pollen trap provides valuable spatial and temporal information of the botanical quality of the environment by means of the biodiversity of this pollen at the time of sampling [40,41]. One current drawback of studies using this method is that the application of pollen traps by scientists to collect pollen loads is often limited to few study sites [2,8,42]. Nevertheless, it remains the most practical direct assessment of pollen diversity.

With this in mind, and to study temporal and spatial pollen diversity throughout Europe, the COLOSS (prevention of honey bee COlony LOSSes, a non-profit bee research association) CSI Pollen (Citizen Scientist Investigation) project was set up with the aim of helping beekeepers to better understand the pollen forage diversity for their bees [43]. In an area as large as Europe, this would be beyond the resources of a few researchers or even several research institutes. Therefore, we chose a citizen science approach involving the help of beekeepers in collecting and recording pollen colors. The involvement of citizen scientists is common in ecological research [44,45,46]. Beekeepers, as a group of citizens with special knowledge and equipment, have been employed in several scientific investigations on bees [47,48,49,50].

A citizen science approach, however, must be both simple and robust with respect to its reliability. Therefore, we chose the number of different colors as a proxy for pollen diversity. Different colors of pollen loads usually represent 1 or 2 main plant species, but they can also be representative of 1 to even 12 different plant species [51]. However, although discrimination of pollen by color is less accurate than palynological identification by light microscopy, it can be used as a simple parameter for pollen diversity [52,53]. In our study, citizen scientists determined pollen diversity by color, which is a rough estimation of the number of pollen species. Firstly, human vision is limited, and secondly, several plant species may produce pollen of the same color, or alternatively, a single plant species may produce several different variations of pollen color [52,54,55,56]. Chromatic assessment therefore probably underestimates the number of pollen types compared to palynological analysis using light microscopy, as demonstrated by Conti et al. [53]. These authors also show that the number of pollen types and the Shannon–Wiener diversity index derived from the chromatic or palynological assessment are significantly correlated, which legitimates the determination of different colors in pollen samples as a simple measure for diversity. The number of different colors identified by citizen scientists was statistically modelled based on whether a minimum amount of pollen was collected or not, season, and habitat type (determined by citizen scientists and according to CORINE land-cover dataset). This will result in information on pollen diversity in different habitats considering different climates and environments.

## 2. Materials and Methods

### 2.1. Sampling and Data Collection by Citizen Scientists

In pilot studies conducted in four countries (Austria, Greece, The Netherlands, and Switzerland) in 2013, we developed and tested a simple protocol for standardized sampling and color discrimination of pollen loads from pollen traps. Volunteer beekeeper citizen scientists were recruited and instructed by national coordinators using an illustrated manual [57]. The citizen scientists in our study were beekeepers who were recruited through talks at beekeeping meetings, articles in beekeeping journals and the internet. To ensure standardized and simultaneous actions by the citizen scientists, all instructions and communications were translated into 17 different languages, and email alerts were sent when sampling was due. They were instructed using a picture manual, and a native speaker expert was assigned for each region or language as a local coordinator to act as a contact person for questions, troubleshooting, support, and feedback. Local coordinators were trained in workshops on how to recruit and instruct citizen scientists. The study was performed throughout the active honey bee season (April–September 2014 and 2015), with samplings every three weeks, nine per year (see Table A1, also for abbreviations used for dates).

The beekeeper citizen scientists were asked to sample up to three non-migratory honey bee colonies in one apiary on the same date. The colonies were numbered one, two, and three and were used unchanged throughout the complete annual sampling period. In some countries where funding was available, citizen scientists were provided with pollen traps, whilst other participants provided their own pollen traps. A pollen trap is a modified hive entrance that causes incoming foragers to lose their corbicular pollen loads into a tray [58]. In the instruction manual, we pointed out the importance of regular cleaning and we recommended the use of disposable paper towels in the sampling tray to avoid contamination of subsequent samples.

Citizen scientists were instructed to sample the incoming pollen loads, ideally of one full day, but more days were permissible if weather conditions were unfavorable, in order to accumulate a minimum amount of pollen. Each sampling was scheduled within a four-day timeframe (Thursday to Sunday), within which the citizen scientists could choose a day suitable for them to activate the traps and then collect pollen samples. The duration of time over which the pollen trap was activated (1 day, 2 days, 3 days, more than 3 days) was submitted together with the pollen color counts.

**Figure A1:**
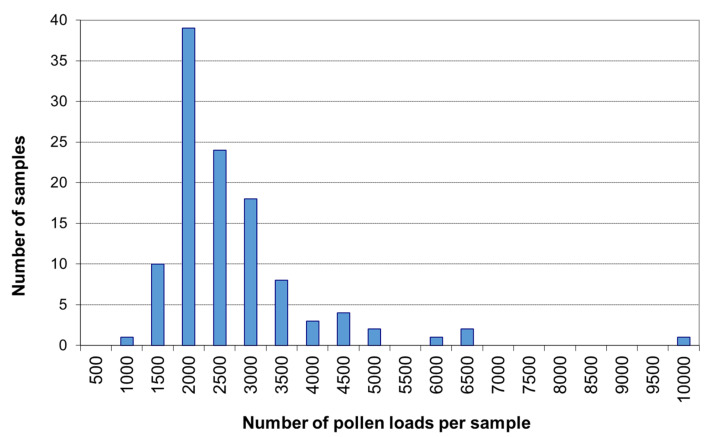
Number of pollen loads per sample that fit in a standard honey jar lid. *n* = 113 samples collected by citizen scientist beekeepers in Spain (excl. Tenerife) and manually counted.

Having obtained the sample, the citizen scientists first determined the mass of the pollen collected per colony. If the pollen harvest per colony was less than 20 g, all the pollen was used for analysis. If the pollen harvest exceeded 20 g, all the pollen was mixed thoroughly, and a subsample of 20 g was taken out for determination of the number of colors. To ensure a comparable amount of pollen, citizen scientists were instructed to use a standard honey jar lid (TO 83) as a measure, because this volume holds roughly 20 g of pollen, representing a median number of 2374 pollen loads (Figure A1). This is more than the minimum required to estimate color diversity. Thompson [59] provides guidelines for estimating proportions of a population belonging to different categories. According to these, a sample of size 510 will result in a 95% probability of all estimated proportions being within 0.05 of the correct proportions, and for our purposes of estimating the number of pollen colors (categories), 510 pollen loads can therefore be considered as giving a 95% chance of a suitably correct assessment of the color diversity. The collected 20 g samples were therefore more than sufficient for the purpose, while being straightforward for the beekeeper to measure.

**Figure 1:**
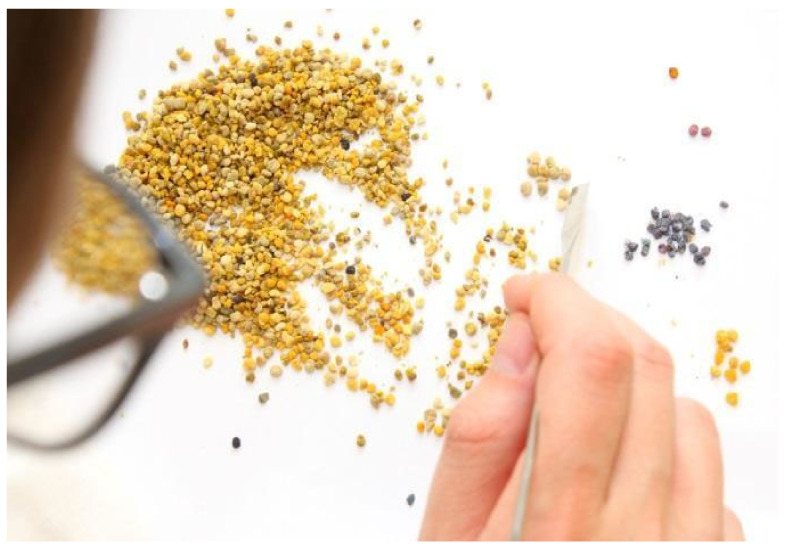
Sorting corbicular pollen loads collected in pollen traps by color using a spatula. Photo: Bernd Niederkofler.

After pollen harvest, the fresh pollen sample was spread on a white piece of paper. Using a paintbrush or other pointed object, pollen loads were separated into distinct different colors (Figure 1) and the number of colors counted according to the following three categories: (a) a “very rare” color (only one or two pollen loads with the same color in the sample); (b) a “rare” color (three to 20 pollen loads with the same color in the sample) and; (c) an “abundant” color in the sample (more than 20 pollen loads with the same color in the sample). Citizen scientists reported the numbers of different colors for each colony and for each sampling period online.

For online data collection, we used the multilingual open-source survey application Limesurvey (Version 1.91+ Build 12416) with standardized text in 17 languages. We invited citizen scientists by email on a Tuesday for sampling in the time window of the following Thursday to Sunday. Each invitation email contained a unique weblink, which allowed the participants to submit their data online in Limesurvey. The unique weblink identified them for later compilation of data in a database. After they had successfully entered their data, the citizen scientists received a confirmation email. If they had not submitted data by the Thursday after a sampling weekend, a single reminder email was automatically sent. Online collection of data closed 4–6 weeks after each sampling date.

All participants were asked once per study season to precisely locate their apiary sites on a Google map embedded in Limesurvey. From this, the latitude, longitude, and altitude of the apiary were obtained for later use. We also asked citizen scientists for a description of nearby habitats. Participants could choose between the following categories: arable/urban/village/grassland/heathland or moorland/salt marsh/deciduous woodland = broad-leaved forest/coniferous woodland/mixed forest/riparian forest and could give multiple answers (any that applied). To keep it simple for the participants, we refrained from precise definitions of the different categories.

### 2.2. Landscape Composition

**Figure 2:**
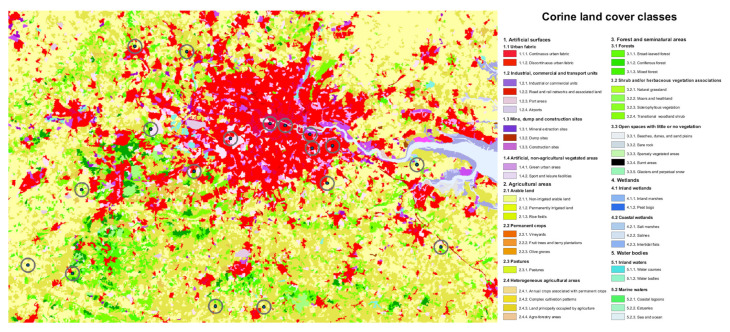
Exact location (point) and 2 km buffers (circles) around sampling sites in the area of greater London, UK. Map shows CORINE land cover classes.

Since the habitat description by the beekeepers represents a rough estimate of the habitats nearby, we additionally used, as verification, spatial information on the number of land-cover types over the sampling area obtained from the CORINE land-cover dataset [60]. The European Environmental Agency provides the coordinate information on the environment (CORINE) land data base, a pan-European land cover/land-use map for non-commercial use. The resolution of the data is 100 × 100 m across Europe. Unfortunately, for Greece no land use data were available for analysis. Landscape structure was assessed over a 2 km radius surrounding each locality to facilitate comparability to other studies [61,62,63,64]. For habitat typology, the CORINE data contains different levels of resolution. We used the level 1 resolution, i.e., the broad definitions of agricultural areas, artificial areas (urban fabric; industrial, commercial, and transport units; mine, dump and construction sites; artificial, non-agricultural vegetated areas), forest and semi natural areas, water bodies, and wetlands to categorize the landscape [65], as this resolution is most comparable to the habitat categories provided by the beekeepers. The analyses were carried out using ArcGIS 10.0 [66]. See Figure 2 for an example.

### 2.3. Statistical Analysis

To investigate the influence of several variables on the number of pollen colors observed, we fitted generalized linear mixed models with a Poisson distribution for the number of colors and using a log link function. The total number of pollen colors (i.e., the sum of abundant, rare, and very rare colors), the number of abundant colors, and the number of rare colors were each used as the dependent variables in separate models. We did not run models with the number of very rare colors as the dependent variable, because variation in the number of very rare colors was too low (i.e., in most cases, the number was between 0 and 1). The dependent variables were all count variables taking values of 0 or more; hence, the Poisson distribution was considered. However, 16 was the maximum reported number for each of these (abundant, rare, very rare), as the number of colors that could be specified was restricted in the questionnaire, meaning 16 codes for 16 or more colors. All models were fitted with R version 3.3.1 [67], using the packages ‘lme4’ [68] and MASS’ [69].

Fixed effects in the full models included the following variables: the latitude, longitude, and altitude of the apiary (continuous variables); the presence of arable land, urban/town, village, grassland, heath/moorland, salt marsh, deciduous woodland, coniferous woodland, mixed forest, and riparian forest as factors with two levels (“yes” and “no”); the number of habitat types surrounding the apiary (range 1–10); the colony (with three levels: “colony 1”, “colony 2”, and “colony 3”); the duration of time when the pollen trap was open (with four levels: “1 day”, “2 days”, “3 days”, and “more than 3 days”); and collection (with two levels: “the required amount of pollen was reached”, and “the required amount of pollen was not reached”). Colony labelling as 1/2/3 was arbitrary but consistent throughout the sampling period. The colony could have been treated as a random effect; however, colony was never found to be a significant effect (see below). The estimation of the fixed effects latitude, longitude, and altitude failed to converge, and these variables had to be rescaled (z-transformed). The results were then interpreted in terms of the number of standard deviations above or below the mean latitude, longitude, or altitude.

Random effects included the ID of the beekeeper—if a beekeeper had more apiaries in the study, more IDs were included—the sampling period (from 1 to 9), the region of the participating beekeeper, and the year of sampling (2014 or 2015), enabling modelling of both years at once (and viewing the two years as representing a larger population of years). For models with rare pollen colors, we added an additional random effect, the ID of the beekeeper per sampling period and colony, to account for over-dispersion.

To test for a possible influence of the habitats as provided by CORINE on pollen diversity, we ran a different set of full models. For these models, we included the following fixed effects: the latitude, longitude, and altitude of the apiary (continuous variables); the proportion of five habitat types provided by CORINE (i.e., artificial surfaces, agricultural areas, forest and semi-natural areas, wetlands, and water bodies); the number of habitat types surrounding the apiaries as stated by the beekeeper (range 1–10); the colony (with three levels: “colony 1”, “colony 2”, and “colony 3”); the duration the pollen trap was open (with four levels: “1 day”, “2 days”, “3 days”, “more than 3 days”); and collection (with two levels: “the required amount of pollen was reached”, and “the required amount of pollen was not reached”). The fixed effects latitude, longitude, altitude, artificial surfaces, agricultural areas, and forest and semi-natural areas failed to converge due to different scaling, and so these variables were rescaled (z-transformed), as above. Random factors included the ID of the beekeeper, the sampling period (from 1 to 9), the region of the participating beekeeper, and the year of sampling. For models with rare pollen colors, we added an additional random factor, the ID of the beekeeper per sampling period and colony, as above, to account for over-dispersion.

Model selection was performed using a forward selection approach [70] and using likelihood ratio tests and the Bayesian information criterion (BIC) as model fit criteria. Fixed effects with significant effects on the dependent variables are shown as confidence interval plots using the package ‘gplots’ [71].

## 3. Results

**Figure 3:**
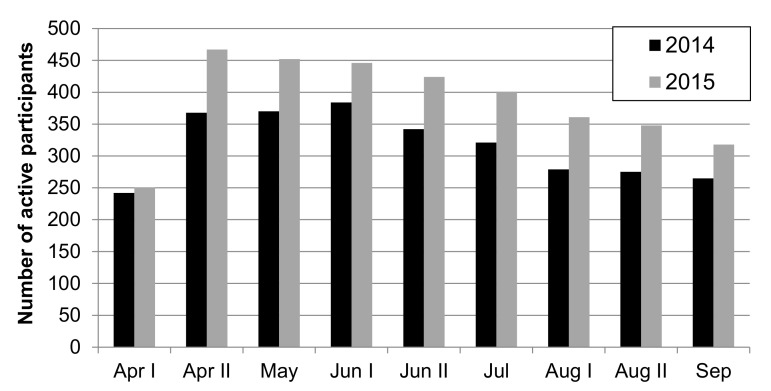
Number of active citizen scientists per sampling in 2014 and 2015.

**Figure 4:**
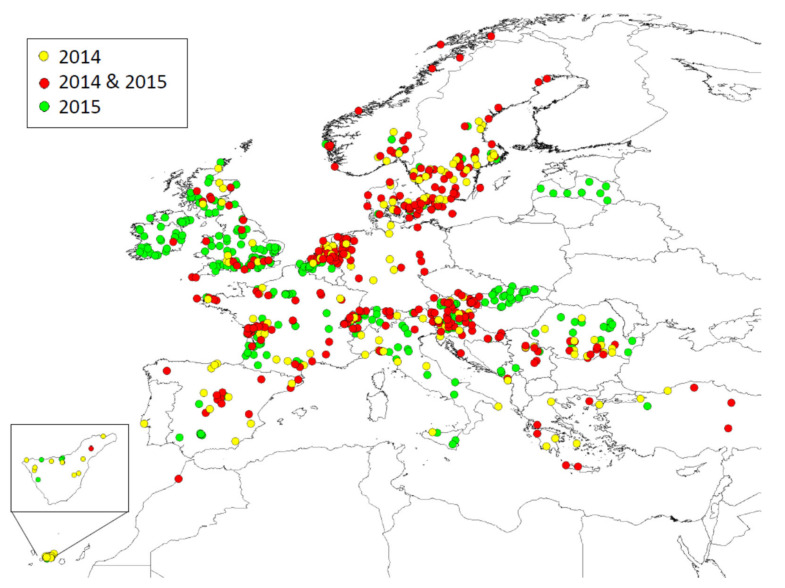
Map showing sampling locations in 2014, 2015, and in both years. Insert shows the island of Tenerife.

Overall, 750 beekeepers from 24 countries participated over the two years. In total, 300 of these beekeepers participated in both years, 165 beekeepers collected pollen in 2014 only (making a total of 465 beekeepers in 2014), and 285 beekeepers collected pollen in 2015 only (making a total of 585 beekeepers in 2015; see Table A2). The participation of citizen scientists (number of active participants) in each sampling of the two years is shown in Figure 3. As we had separate regional coordinators for Tenerife, England, Northern Ireland, Scotland, and Wales, we here present results from 28 different regions. Our sampling area covered 24 regions in 2014 and 27 regions in 2015. The location of the apiaries of participants is shown in Figure 4. We received records of a total of 8094 sampling events in 2014 and a total of 9823 in 2015.

**Figure 5:**
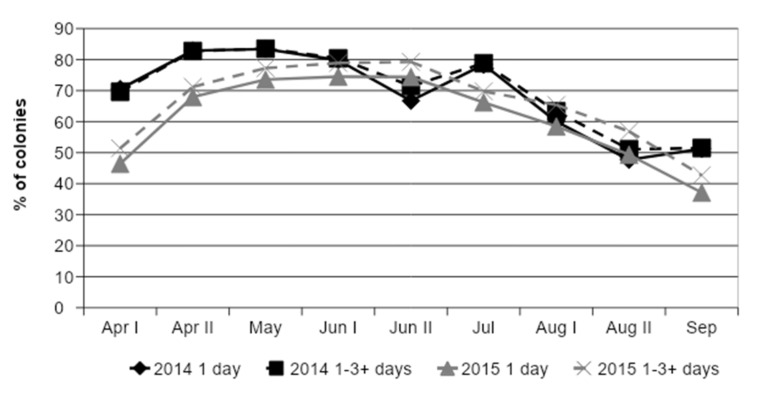
Percentage of honey bee colonies that collected the required amount of 20 g pollen loads throughout the season. Data from two years and trapping duration (1 day) or 1 to 3 or more days (1–3+ days) are shown. *n* = 209–1241 colonies sampled by citizen scientists per data point.

In 2014, 72.2% (*n* = 7516) of the collected samples met the required 20 g and 68.3% in 2015 (*n* = 9048). The percentage of successful pollen trappings (bees collected more than 20 g) followed a seasonal pattern. This seasonal pattern was also observed for samplings collected exactly within one day and sample collections lasting between one or three and more days (Figure 5). More than half of the samples (58.2%) were collected by trapping pollen on one day, 20.5% for two days, 16.8% for three days, and 4.5% of the samples were obtained by trapping pollen for more than three days (*n* = 16,564 colonies).

**Figure 6:**
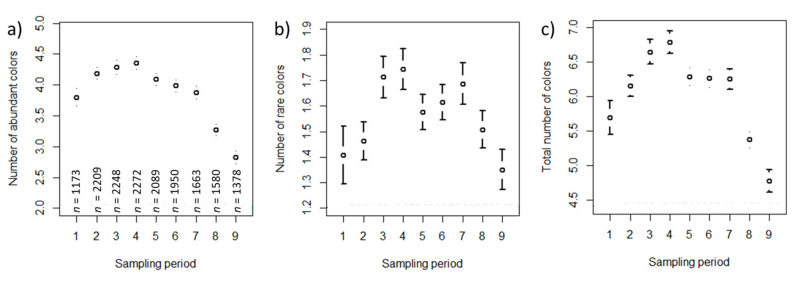
Confidence interval plots (mean and 95% CI) for number of colors per sampling period. (**a**) number of abundant colors per sampling period; (**b**) number of rare colors per sampling period; (**c**) total number of colors per sampling period. Sample sizes in (**a**) apply for (**b**) and (**c**) too.

The mean numbers of abundant, rare, and total colors per sampling period also followed a seasonal pattern and are shown in Figure 6a–c.

### 3.1. Models with Habitat Types According to the Citizen Scientists’ Specification

The fitted models are based on the samples provided by 727 beekeepers (16,564 samples for abundant and rare colors, 16,562 samples for the total number of colors). Data from 23 beekeepers had to be excluded from analysis due to missing or illogical information with respect to the location and habitat types surrounding the apiary.

The number of abundant colors was significantly affected by collection (i.e., whether the required amount of 20 g pollen was reached or not), the habitat (i.e., whether arable land surrounded the apiary or not), and the duration the trap was open (Table 1). As we used the log link, the parameter estimates were exponentiated to interpret changes in the response scale (i.e., actual number of abundant colors). The intercept describes the expected number of abundant colors (on the log scale) if the required amount of pollen was reached, no arable land surrounded the apiary, and the trap was open for one day (the baseline settings in the model). The estimated number of abundant colors for this “reference” sample (intercept) was approximately 4.2 (last column, Table 1).

**Figure 7:**
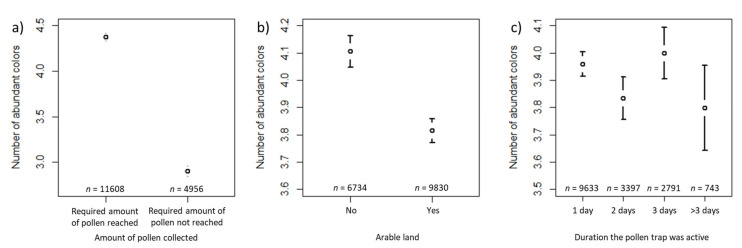
Confidence interval plots for number of abundant colors as dependent variable and sampling amount and duration and habitat types according to the beekeepers’ specification as fixed effects. Mean (and 95% CI) number of abundant colors in samples where (**a**) the required amount of pollen was reached vs. samples where the required amount was not reached; (**b**) arable land vs. no arable land surrounded the apiary and: (**c**) the pollen trap was open for different durations.

The number of abundant colors decreased by a factor of 0.703 (Table 1) for the samples where the required amount of pollen was not reached compared to samples where the required amount of pollen was reached (Figure 7a). In samples for which arable land surrounded the apiary, the number of abundant colors decreased by a factor of 0.940 (Table 1) compared to samples where no arable land surrounded the apiary (Figure 7b). The number of abundant colors did not differ significantly between samples collected for one day and samples collected for two or more than three days. However, the number of abundant colors increased by a factor of 1.036 in samples collected for three days, compared to the samples collected for one day (Table 1, Figure 7c). The estimated variance components of the random effects are also shown in Table 1. Of the random effects, beekeeper ID had the largest effect, and year was the smallest.

**Figure 8:**
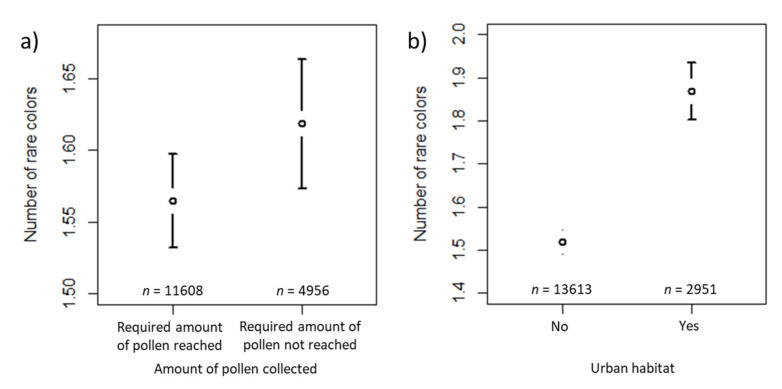
Confidence interval plots for number of rare colors as a dependent variable and habitat types according to the beekeepers’ specification as a fixed effect. Mean (and 95% CI) number of rare colors in samples where: (**a**) the required amount of pollen had been reached vs. samples where the required amount had not been reached; (**b**) urban habitat vs. rural habitat surrounded the apiary.

The number of rare colors was significantly affected by collection (i.e., whether the required amount of 20 g pollen had been reached or not) and habitat (i.e., whether urban habitat surrounded the apiary or not; Table 2). The number of rare colors for a “reference” sample (intercept) reaching the required amount of pollen and not having urban habitat type in the surroundings of the apiary was about 1.2 (last column, Table 2). The number of rare colors increased by a factor of 1.070 for the samples where the required amount of pollen had not been reached (Table 2) compared to the samples where the required amount of pollen had been reached (Figure 8a). In samples where urban habitat surrounded the apiary, the number of rare colors increased by a factor of 1.206 (see Table 2) compared to the samples where the apiary was not surrounded by urban habitat (Figure 8b). The estimated variance components of the random effects are shown in Table 2. Of the random effects, beekeeper ID was the largest effect, followed by ID of the beekeeper per sampling period and colony, whereas the other random effects were negligible.

**Figure 9:**
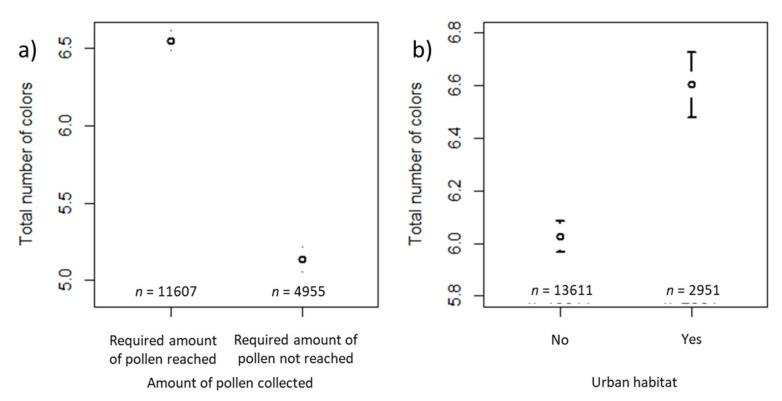
Confidence interval plots for the total number of colors as a dependent variable and habitat types according to the beekeepers’ specification as a fixed effect. Mean (and 95% CI) total number of colors in samples where: (**a**) the required amount of pollen had been reached vs. samples where the required amount had not been reached; (**b**) urban habitat vs. rural habitat surrounding the apiary.

The total number of colors was significantly affected by collection (i.e., whether the required amount of 20 g pollen had been reached or not) and habitat (i.e., whether urban habitat surrounded the apiary or not; Table 3). The estimated total number of colors for a “reference” sample reaching the required amount of pollen and not having urban habitat type in the surroundings of the apiary was 6.0 (last column, Table 3). The total number of colors decreased by a factor of 0.821 for the samples for which the required amount of pollen had not been reached (Table 3) compared to those for which the required amount of pollen had been reached (Figure 9a). For samples in which urban habitat surrounded the apiary, the total number of colors increased by a factor of 1.101 (Table 3) compared to those in which the apiary was not surrounded by urban habitat (Figure 9b). The variance components of the random effects are also shown in Table 3. Again, ID of the beekeeper was the largest random effect, and year was the smallest.

### 3.2. Models with Habitat Proportion According to the CORINE Data

The fitted models are based on the samples provided by 717 beekeepers from 26 different regions (16,324 samples for abundant and rare colors; 16,322 samples for the total number of colors). Data from 33 beekeepers were excluded from analysis due to missing or illogical information with respect to the location of the apiary.

**Figure 10:**
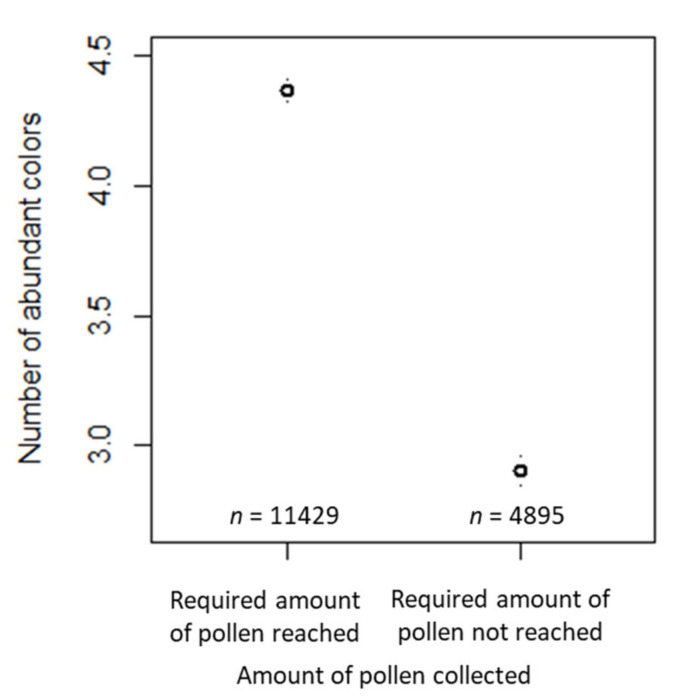
Confidence interval plot for number of abundant colors as a dependent variable and habitat proportions according to the CORINE data as a fixed effect. Mean (and 95% CI) number of abundant colors in samples where the required amount of pollen had been reached vs. samples where the required amount had not been reached.

The number of abundant colors was significantly affected by the collection success (Table 4). For the model using the habitat proportions according to CORINE (Table 4), the estimated number of abundant colors for a “reference” sample, in this case reaching the required amount of pollen, was around 4.0. For samples for which the required amount of pollen had not been reached, the number of abundant colors decreased by a factor of 0.705 (Table 4) compared to those for which the required amount of pollen had been reached (Figure 10). This was very similar to the result for the model with habitat type specified by the beekeepers. The estimated variance components of the random effects are shown in Table 4. ID of the beekeeper was the largest random effect, and year was the smallest.

**Figure 11:**
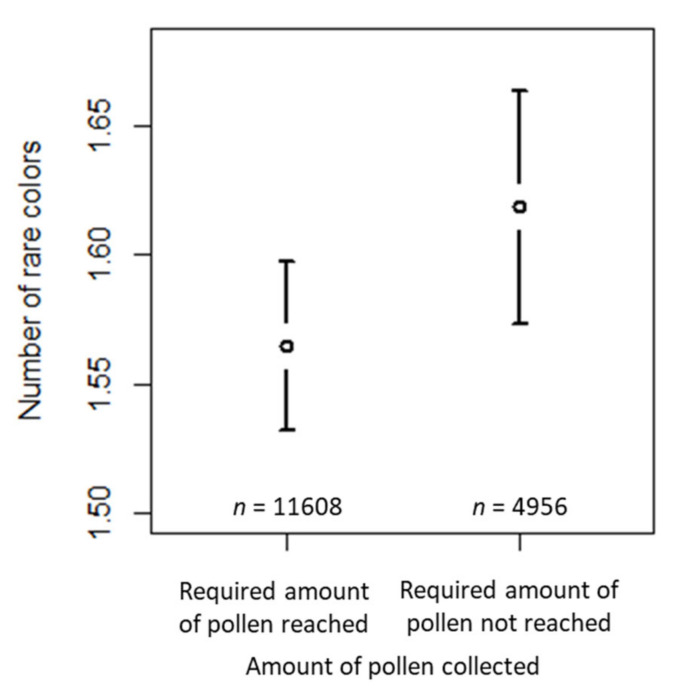
Confidence interval plots for number of rare colors as dependent variable and habitat proportions according to the CORINE data as a fixed effect. Mean (and 95% CI) number of rare colors in samples where the required amount of pollen had been reached vs. samples where the required amount had not been reached.

The number of rare colors was significantly affected by collection (i.e., whether the required amount of 20 g pollen was reached or not) and habitat (i.e., whether artificial surfaces (z-transformed) surrounded the apiary or not; Table 5), as with the finding of the corresponding model, including habitat type specified by the beekeepers. The estimated number of rare colors for a “reference” case reaching the required amount of pollen and having the mean proportion of artificial surfaces in our sample was about 1.3. The mean proportion (expressed as a percentage) of artificial surfaces in our sample was 17.6%, and the standard deviation in the proportion was 24.4%. An exponent of 1.101 for artificial surfaces (z-transformed) meant that every increase (decrease) of 10% in the percentage of artificial surfaces above (below) the mean of 17.6% resulted in an increase (decrease) in the number of rare colors by a factor of 1.101 raised to the power 10/24.4, i.e., 1.040. For the samples for which the required amount of pollen had not been reached, the number of rare colors increased by a factor of 1.069 (Table 5) compared to those for which the required amount of pollen had been reached (Figure 11). The estimated variance components of the random effects are shown in Table 5. Again, ID of the beekeeper was the largest random effect, followed by ID of the beekeeper per sampling period and colony, and year was the smallest.

**Figure 12:**
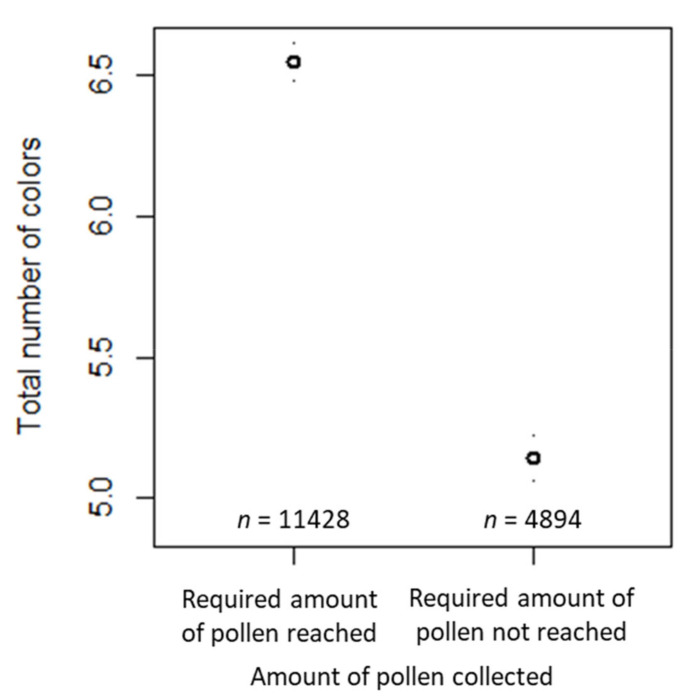
Confidence interval plots for the total number of colors as a dependent variable and habitat proportions according to the CORINE data as a fixed effect. Mean (and 95% CI) total number of rare colors in samples where the required amount of pollen had been reached vs. samples where the required amount had not been reached.

The total number of colors was significantly affected by collection (i.e., whether the required amount of 20 g pollen had been reached or not) and habitat (i.e., whether artificial surfaces (z-transformed) surrounded the apiary or not; Table 6). The estimated total number of colors was about 6.1 for a “reference” case. In this model, the reference case reached the required amount of pollen and had the mean proportion of artificial surfaces in our sample. For the samples for which the required amount of pollen was not reached, the total number of colors decreased by a factor of 0.823 (Table 6) compared to those for which the required amount of pollen was reached (Figure 12). The mean proportion of artificial surfaces was 17.6%, and the standard deviation was 24.4%. An exponent of 1.050 for artificial surfaces (z-transformed) meant that every increase in the percentage of artificial surfaces of 10% above (below) the mean of 17.6% resulted in an increase (decrease) in the total number of different colors by a factor of 1.050 raised to the power of 10/24.4, i.e., 1.020. The estimated variance components of the random effects are shown in Table 6. Again, the largest random effect was for beekeeper ID and the smallest for year.

In summary, collection was an important fixed effect in all the final models. Urban habitats or proportion of artificial surfaces were also significant in the models for number of rare colors or total number of colors. In addition to collection, arable land and the duration for which the traps were open were significant in the model using beekeeper-specified habitat type. For the random effects, the random effects involving beekeeper ID were the largest, with other effects, especially year, being comparatively smaller. The size of the effects was similar in the models for total number of colors and number of abundant colors, and also when comparing the models using the CORINE data and the models using beekeeper-specified habitat type. The very small variance components indicated low group-level variation for region, sampling period, and year.

## 4. Discussion

In the approximately 18,000 pollen samples, we found, on average, a total number of six colors, of which four were abundant and one was rare (Figure 6). The variation in the number of colors (in the range visible to the human eye) is therefore lower than the variability usually reported from palynological studies. A pollen sample typically contains many more pollen morphotypes that can be discriminated, although the number of those with a proportion of more than 1% of a sample can be estimated to be between 10 and 20 [1,3,53,72]. Color diversity of the pollen samples was significantly influenced by the amount of pollen collected (i.e., whether the required amount of 20 g of pollen had been reached or not) and the habitat surrounding the apiary.

In our study, it was of great importance that all citizen scientists worked with the same, standardized amount of pollen, because the species diversity could be affected by the amount of pollen analyzed. Palynological studies using light microscopy usually analyze just a few grams of pollen [1,2,53]. Citizen scientists in our study were instructed to determine whether bees had collected enough pollen to fill an inverted honey jar lid, a tool easily at hand for a beekeeper. This container holds about 20 g or circa 2000 pollen loads (Figure A1). As expected, the amount of pollen sampled and analyzed significantly affects the number of abundant and rare pollen colors in the sample according to all models. Samples with lower amounts as described above had more rare pollen colors, but at the expense of the number of abundant pollen colors (Figure 7, Figure 8, Figure 9, Figure 10, Figure 11 and Figure 12). Whether the required amount of pollen was reached could also be linked to the likelihood of colonies gathering enough pollen for analysis throughout the season. The pollen needs of a honey bee colony are largely determined by intra-colonial factors, such as the amount of open brood in the colonies, pollen stores, and colony strength [73]. Furthermore, external factors such as weather or competition with other colonies may also affect the amount of pollen collected [4]. Most pollen samples collected by bees in both years reached the required amount of pollen for determination of colors, but successful pollen collection was more likely in samplings between the end of April and July. Although Figure 5 shows the likelihood that bees collected the required amount of pollen as a function typical for seasonal effects, with a steep increase from April to May and a gradual decrease after sampling in late July, we must be careful regarding direct conclusions about the environmental abundance of pollen at our sampling sites. Firstly, pollen traps were not standardized in our study and probably varied greatly in their efficiency to harvest pollen [58], and secondly, other factors apart from environmental availability also influence pollen collection. The seasonal effect described here was, therefore, more related to the amount of brood in the colony.

In contrast to controlled studies, the citizen scientists in our study did not locate colonies in specially chosen landscapes but instead used their established apiaries. To evaluate trends in landscape composition on the diversity of pollen colors, we asked citizen scientists to describe the habitat surrounding their colonies and additionally applied land cover composition from the CORINE dataset. The generalized linear models, including both types of habitat information, demonstrate a positive effect of anthropogenic habitats on the number of rare pollen colors and the total number of pollen colors (‘urban’ according to the citizen scientists’ specification, Table 2 and Table 3, Figure 8 and Figure 9, and ‘artificial surfaces’ according to the CORINE dataset, Table 5 and Table 6). This is the first evidence from a large-scale study that urban habitats provide more pollen diversity to honey bees. Previously, this was demonstrated for a few study sites only from the USA [42,74] and one study from southeast England [75]. The supply of sufficient and diverse pollen is considered a key feature of honey bee health [32,76,77,78]. Habitat and landscape composition are closely linked to pollen sources, but only minor effects of landscape on pollen diversity were reported by a study in German agricultural landscapes [64]. This might be due to the efficiency of honey bees to locate and communicate worthwhile pollen sources within 2 km or even more distantly [61,79]. Gardens, in contrast to plantations and forests, provide good nectar and pollen sources for bee colonies [80], and urban areas have been found to be spots of high plant- and flower-visiting insect species richness [62,81]. Additionally, urban areas in Denmark were previously shown to yield higher colony productivity [82]. Sponsler and Johnson [23] could also distinguish the productivity and other characteristics of honey bee colonies from urban areas in contrast to forest, grassland, or crop areas, but found that food accumulation decreased significantly with increasing urban land cover. We suggest that higher pollen diversity is available in urban areas, which is not incompatible with the finding of mass nectar supplies being more prevalent in crop areas. Arable land, as specified by citizen scientists, had a decreasing effect on the number of abundant pollen colors (Table 1, Figure 7), but this could not be confirmed by our model based on the CORINE data. The recording of more rare pollen and less abundant pollen if the 20 g per sampling was not met indicated that there are no or very few mass pollen sources in a particular location, forcing the bees to collect less profitable pollen sources.

Season influenced the color diversity of pollen samples and was therefore included as a random effect in all the final models. The low number of abundant colors found in pollen samples taken in late August or September (Figure 6) reflects the often-observed lack of diverse pollen sources late in the season. This supports palynological analysis from Ireland, which found the diversity of pollen sources for honey bee colonies was the highest in June and July, with decreasing diversity towards September [2]. Similar trends showing the highest diversity of pollen collected by honey bee colonies in spring/early summer have been demonstrated for Scotland [1], Greece [1,83], Turkey [84], France [33,85], and Austria (using the samples collected by citizen scientists in this study) [86]. Our results based on pollen color diversity therefore support the suggested strong impact of season on diversity of pollen sources collected by honey bee colonies [64].

Many citizen science studies indicate that, when given proper training and materials, volunteers can collect data comparable to data collected by professional scientists [44,87]. The limits of citizen science are reached when it comes to expert knowledge, for example regarding the taxonomy of bee species [88]. Our study has shown that beekeeper citizen scientists can be recruited and trained over a wide geographical area to conduct a coordinated experiment much larger than individual researchers could ever achieve. Most citizen scientists participated in this project for two sampling seasons. All of them supplied their own honey bee colonies, and many also supplied their own pollen traps. The voluntary work contribution of the 750 individual citizen scientists amounted to an estimated 6000 h, in which they collectively sampled and sorted almost 18,000 pollen samples. The protocol chosen was deliberately restricted to one simple parameter (pollen load color), which required neither a scientific background nor specialist equipment to be measured. Figure 3 shows the participation of citizens in CSI Pollen, which was low in the first samplings each year, probably due to unfavorable weather conditions or logistical problems in some countries. Similarly, towards the tail-end of the study, participation slightly dwindled, as is often reported for long running citizen science projects [89]. To minimize this phenomenon, national coordinators were regularly in touch with participants through lectures, competitions, multimedia, and personal contact. Volunteers’ participation has been open to other audiences than beekeepers in the strict sense, such as municipalities or schools possessing honey bee colonies. During the investigation, many interactions between researchers and citizen scientists occurred, and both profited from the study [90]. The impact of our study has not only highlighted the importance of landscape and season on pollen diversity for honey bee nutrition among participating beekeepers and the beekeeping community in general, but, in addition, has brought awareness to the larger public of the question of the linkage of landscape and biodiversity, a major current topic of global debate.

Our findings show that honey bees at different locations throughout much of Europe collect about four different abundant colors of pollen, with comparable low variance (Table 1, Table 2, Table 3, Table 4, Table 5 and Table 6). The polylectic honey bee is hence exquisitely adapted to collect a comparable diversity of pollen in different vegetation zones from north to south in Europe [37]. This raises the question of whether bees can perceive and regulate the diversity of pollen at the colony level, or instead randomly collect pollen from those plants available in the environment. Investigations of floral choice of honey bees suggest that honey bees do not sample the environment representatively, in spring visiting only 11% of the flowering plants in a botanical garden [5]. Dimou and Thrasyvoulou [3] found that bees collected pollen from only half of the flowering taxa present within 1 km around an apiary, but also found pollen from flowers not recorded by the observers. They also reported a positive correlation between the number of blooming taxa and the number of pollen types collected. Interesting findings of pollen foraging ecology are also available on another generalist social bee species belonging to the tribe Meliponini. It was suggested that this stingless bee species maximizes pollen diversity intake in resource diverse habitats [91,92]. No such predication can so far be made on honey bees, and further research on this topic is recommended.

Our findings do not quantify the foraging distance of honey bees required to collect the pollen diversity recorded in our study. Honey bees prefer to search within a short distance of their nest but can fly further to seek pollen sources in times of low pollen abundance or in pursuit of especially attractive flowering crops or wild plants [93]. Seasonal variation in foraging distance has been demonstrated, and accordingly, distance is discussed as a proxy for resource availability [61,62,79]. In landscapes with low pollen diversity, pollen foragers compensate for lower forage availability by increasing their foraging range to maintain pollen amount and diversity [64,94]. This might be more important in pollen foraging compared to nectar foraging, but the effect on the amount of forage collected is not yet clear [95].

## 5. Conclusions

CSI Pollen was the largest study to investigate pollen diversity for honey bee colonies. We demonstrated that citizens can be trained to take samples and make a simple analysis, which can be used to study pollen supply and diversity on a landscape scale. In some countries, citizen scientists could send pollen samples to laboratories for further analysis [53,86]. Our main findings suggest higher diversity in artificial, non-agricultural surfaces. The results from this study support the importance of diverse forage availability for honey bees and other wild pollinators, and participation has hopefully increased awareness of both citizen scientists and the general public for this topic.

## Figures and Tables

**Table 1:** Estimated model coefficients for the model with number of abundant colors as the dependent variable and fixed effects including habitat types as stated by the beekeepers.

Fixed Effects	Factor Level	Estimate	Std. Error	*p*-Value	Exp (Estimate) ^1^
Intercept	-	1.4259	0.0471	<2 × 10^−16^ ***	4.1616
Collection (Reference category: Required amount of pollen reached)	Required amount not reached	−0.3518	0.0107	<2 × 10^−16^ ***	0.7034
Arable land (Reference category: Arable land No)	Arable land Yes	−0.0619	0.0260	0.0170 *	0.9400
Duration the trap was active (Reference category: Trap open 1 day)	Trap open 2 days	−0.0117	0.0128	0.3615	0.9883
	Trap open 3 days	0.0356	0.0162	0.0285 *	1.0362
	Trap open > 3 days	−0.0049	0.0268	0.8556	0.9951
Random effects		Variance			
ID beekeeper	-	0.0957	-	-	-
Region	-	0.0049	-	-	-
Sampling period	-	0.0096	-	-	-
Year	-	0.0010	-	-	-

^1^ denotes incidence rate ratio (IRR). * indicates a significant effect with *p*-value such that 0.01 < *p* ≤ 0.05, *** indicates a highly significant effect with *p* ≤ 0.001.

**Table 2:** Estimated model coefficients for the model with number of rare colors as the dependent variable and fixed effects including habitat types as stated by the beekeepers.

Fixed Effects	Factor Level	Estimate	Std. Error	*p*-Value	Exp (Estimate) ^1^
Intercept	-	0.2150	0.0452	1.95 × 10^−6^ ***	1.2399
Collection (Reference category: required amount of pollen reached)	Required amount not reached	0.0673	0.0173	0.0001 ***	1.0696
Urban (Reference category: urban no)	Urban yes	0.1870	0.0563	0.0009 ***	1.2056
Random effects		Variance			
ID beekeeper per sampling period per colony	-	0.1276	-	-	-
ID beekeeper	-	0.2821	-	-	-
Region	-	0.0073	-	-	-
Sampling period	-	0.0069	-	-	-

^1^ denotes incidence rate ratio (IRR). *** indicates a highly significant effect with *p* ≤ 0.001.

**Table 3:** Estimated model coefficients for the model with the total number of different colors as the dependent variable and fixed effects including habitat types as stated by the beekeepers.

Fixed Effects	Factor Level	Estimate	Std. Error	*p*-Value	Exp (Estimate) ^1^
Intercept	-	1.7986	0.0405	<2 × 10^−16^ ***	6.0410
Collection (Reference category: required amount of pollen reached)	Required amount not reached	−0.1968	0.0084	<2 × 10^−16^ ***	0.8213
Urban (Reference category: urban no)	Urban yes	0.0961	0.0316	0.0024 **	1.1009
Random effects		Variance			
ID beekeeper	-	0.0929	-	-	-
Region	-	0.0045	-	-	-
Sampling period	-	0.0073	-	-	-
Year	-	0.0008	-	-	-

^1^ denotes incidence rate ratio (IRR). ** indicates a more significant effect with 0.001 < *p* ≤ 0.01; *** indicates a highly significant effect with *p* ≤ 0.001.

**Table 4:** Estimated model coefficients for the model with number of abundant colors as the dependent variable and fixed effects including habitat proportions according to the CORINE data.

Fixed Effects	Factor Level	Estimate	Std. Error	*p*-Value	Exp (Estimate) ^1^
Intercept	-	1.3931	0.0451	<2 × 10^−16^ ***	4.0273
Collection (Reference category: required amount of pollen reached)	Required amount not reached	−0.3492	0.0108	<2 × 10^−16^ ***	0.7052
Random effects		Variance			
ID beekeeper	-	0.0977	-	-	-
Region	-	0.0052	-	-	-
Sampling period	-	0.0097	-	-	-
Year	-	0.0010	-	-	-

^1^ denotes incidence rate ratio (IRR). *** indicates a highly significant effect with *p* ≤ 0.001.

**Table 5:** Estimated model coefficients for the model with number of rare colors as the dependent variable and fixed effects including habitat proportions according to the CORINE data.

Fixed Effects	Variable or Factor Level	Estimate	Std. Error	*p*-Value	Exp (Estimate) ^1^
Intercept	-	0.2532	0.0476	1.05 × 10^−7^ ***	1.2881
Artificial surfaces scaled (Reference value: mean proportion of artificial surfaces)	Artificial surfaces	0.0966	0.0216	7.36 × 10^−6^ ***	1.1014
Collection (Reference category: required amount of pollen reached)	Required amount not reached	0.0668	0.0174	0.0001 ***	1.0691
Random effects		Variance			
ID beekeeper per sampling period per colony	-	0.1280	-	-	-
ID beekeeper	-	0.2754	-	-	-
Region	-	0.0102	-	-	-
Sampling period	-	0.0074	-	-	-
Year	-	0.0007	-	-	-

^1^ denotes incidence rate ratio (IRR). *** indicates a highly significant effect with *p* ≤ 0.001.

**Table 6:** Estimated model coefficients for the model with the total number of different colors as the dependent variable and fixed effects including habitat proportions according to the CORINE data.

Fixed Effects	Variable or Factor Level	Estimate	Std. Error	*p*-Value	Exp (Estimate) ^1^
Intercept	-	1.8153	0.0414	<2 × 10^−16^ ***	6.1429
Collection (Reference category: required amount of pollen reached)	Required amount not reached	−0.1949	0.0084	<2 × 10^−16^ ***	0.8229
Artificial surfaces scaled (Reference value: mean proportion of artificial surfaces)	Artificial surfaces	0.0486	0.0122	6.47 × 10−5 ***	1.0498
Random effects		Variance			
ID beekeeper	-	0.0925	-	-	-
Region	-	0.0047	-	-	-
Sampling period	-	0.0076	-	-	-
Year	-	0.0009	-	-	-

^1^ denotes incidence rate ratio (IRR). *** indicates a highly significant effect with *p* ≤ 0.001.

## Data Availability

The data are available from the corresponding author upon request.

## Appendix A

**Table A1:** Dates for sampling honey bee collected pollen by citizen scientists.

Abbreviation	Sampling Dates 2014	Sampling Dates 2015
April I	3–6 April	2–5 April
April II	24–27 April	23–26 April
May	15–18 May	14–17 May
June I	5–8 June	4–7 June
June II	26–29 June	25–28 June
July	17–20 July	16–19 July
August I	7–10 August	6–9 August
August II	28–31 August	27–30 August
September	18–21 September	17–20 September

**Table A2:** Number of participating citizen science beekeepers from the different regions in the two years.

Country/Region	Number of Participants 2014	Number of Participants 2015
Austria	55	61
Belgium	0	15
Croatia	8	9
Denmark	31	29
England	16	58
France	48	72
Germany	15	9
Greece	9	5
Ireland	1	24
Italy	15	20
Latvia	0	8
Montenegro	3	4
Morocco	1	1
Netherlands	55	42
Northern Ireland	0	6
Norway	19	21
Portugal	1	0
Romania	22	29
Scotland	11	24
Serbia	9	9
Slovakia	0	21
Slovenia	8	7
Spain	23	22
Sweden	72	47
Switzerland	19	27
Tenerife	10	5
Turkey	12	4
Wales	2	6
Total	465	585
